# Mechanical Reperfusion Following Prolonged Global Cerebral Ischemia Attenuates Brain Injury

**DOI:** 10.1007/s12265-020-10058-9

**Published:** 2020-07-17

**Authors:** Rickard P. F. Lindblom, Thomas Tovedal, Bo Norlin, Lars Hillered, Elisabet Englund, Stefan Thelin

**Affiliations:** 1 Department of Cardiothoracic Surgery and Anesthesia, Uppsala University Hospital, SE-751 85 Uppsala, Sweden; 2 Department of Surgical Sciences, Section of Thoracic Surgery, Uppsala University, Uppsala, Sweden; 3 Department of Surgical Sciences, Section of Anesthesiology and Intensive Care, Uppsala University, Uppsala, Sweden; 4 Department of Neuroscience, Neurosurgery, Uppsala University, Uppsala, Sweden; 5 Department of Clinical Sciences, Lund University, Lund, Sweden

**Keywords:** Global cerebral ischemia, Reperfusion, Mechanical circulation

## Abstract

Previous experiments demonstrated improved outcome following prolonged cerebral ischemia given controlled brain reperfusion using extracorporeal circulation. The current study further investigates this. Young adult pigs were exposed to 30 min of global normothermic cerebral ischemia, achieved through intrathoracic clamping of cerebral arteries, followed by 20 min of isolated mechanical brain reperfusion. Leukocyte-filtered blood was delivered by a roller-pump at fixed pressure and flow. One experimental group additionally had a custom-made buffer solution delivered at 1:8 ratio with the blood. Hemodynamics including intracranial pressure were monitored. Blood gases were from peripheral arteries and the sagittal sinus, and intraparenchymal brain microdialysis was performed. The brains were examined by a neuropathologist. The group with the added buffer showed lower intracranial pressure as well as decreased intraparenchymal glycerol and less signs of excitotoxicity and ischemia, although histology revealed similar degrees of injury. A customized mechanical reperfusion improves multiple parameters after prolonged normothermic global cerebral ischemia.

**Electronic supplementary material:**

The online version of this article (10.1007/s12265-020-10058-9) contains supplementary material, which is available to authorized users.

**Graphical Abstract:**
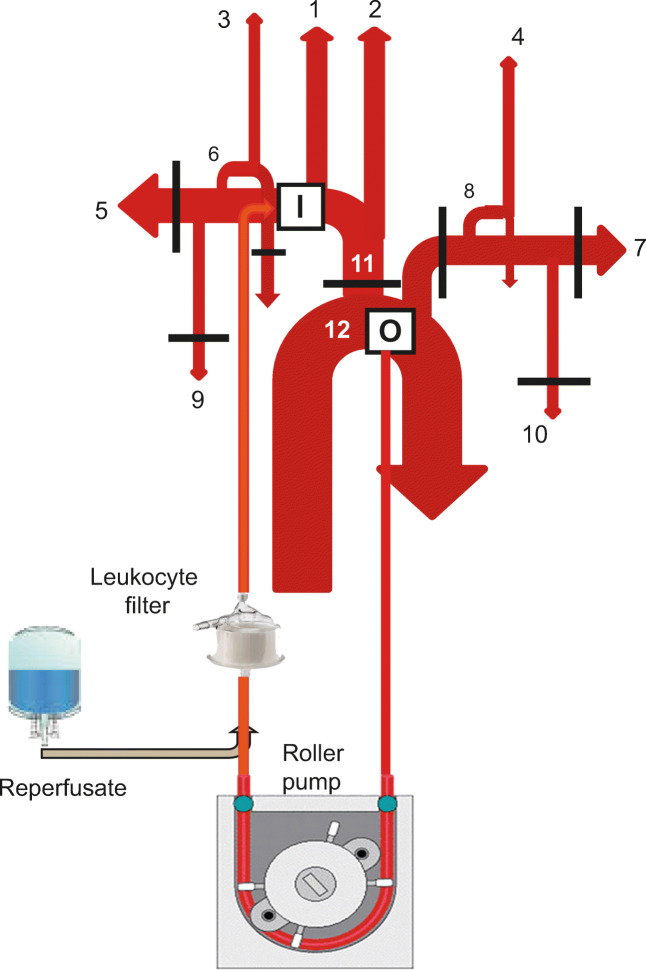
The current study investigates if it possible to improve neurological outcomes following prolonged global brain ischemia. The results indicate that a customized mechanical reperfusion protocol can attenuate neurological injury.

## Introduction

With the introduction of extracorporeal cardiopulmonary resuscitation (E-CPR), the boundaries of cardiac resuscitation are pushed. After conventional CPR, results are hampered by poor neurological outcome even if the circulation can be restored [1]. The same holds to be true following E-CPR, where only 15–20% favorable outcome is achieved, defined as survival with acceptable neurological status [2, 3].

In a series of large animal experiments, Allen et al. demonstrated that the brain could be salvaged even after as long as 30 min of warm, global cerebral ischemia [4–7]. This was achieved through an elaborate controlled reperfusion protocol, using extracorporeal circulation and modified blood/reperfusion solutions delivered specifically to the brain at a controlled flow and pressure. Recent findings demonstrated restoration of neuronal activity ex vivo after as long as 4 h after decapitation [8]. The conclusions were that it was not the ischemia per se, but rather the uncontrolled reperfusion, which occurs once the circulation is reestablished to the ischemic brain that manifests tissue damage. Accordingly, reperfusion damage has been shown in postcardiac arrest encephalopathy in humans [9, 10]. Ischemic-reperfusion injury, however, is a complex process and a multitude of pathways are activated.

In another experiment, pigs exposed to 20 min of ventricular fibrillation followed by direct initialization of a modified E-CPR protocol demonstrated good results with regard to neurologic recovery [11]. This was achieved using hypothermic reperfusion via extracorporeal circulation, where the priming was hyperosmolar, with human albumin, mannitol, citrate, and magnesium. However, no detailed declaration of contents of the priming solution was provided.

We have also studied the effect of mechanical reperfusion following global cerebral ischemia, experiments similar to that of Allen et al. We saw no clear benefit of controlled cerebral reperfusion following 30 min of global ischemia using solely leukocyte-filtered blood [12], compared with no controlled reperfusion. The discrepancies between our and Allen et al. studies urged us to pursue the issue further. The next step toward increased understanding was to focus on the composition of reperfusion solution. In the current study, we compared the results between reperfusing the ischemic brain with a modified reperfusate added to leukocyte-filtered blood, to reperfusion with leukocyte-filtered blood only.

## Materials and Methods

### Ethical Permit

The experiments were performed according to the Uppsala Ethics Committee for Animal Research under permit number C12/13.

### Animals

All animals received humane care in compliance with the European Convention on Animal Care and ARRIVE guidelines. Twenty-two pigs (Swedish country breed, 43.3 ± 2.5 kg) were included. The animals were acquired from a local farmer and transported to the operation facility on the morning of the experiment, one per day. Five pigs were included in the sham group, 7 in the controlled reperfusion group (StRep that received only leukocyte-filtered blood, partially described before [12]), and 10 in the modified controlled reperfusion group (MoRep). Four animals were excluded (1 StRep; died perioperatively, subclavian perforation, 3 MoRep; 2 died perioperatively due to circulatory failure, 1 failure to lower calcium/uncertain effect of reperfusate).

### Anesthesia, General Preparations, and Euthanasia

See supplementary information (SI) and [12].

### Surgery and Isolation of Cerebral Blood Flow

**Fig. 1:**
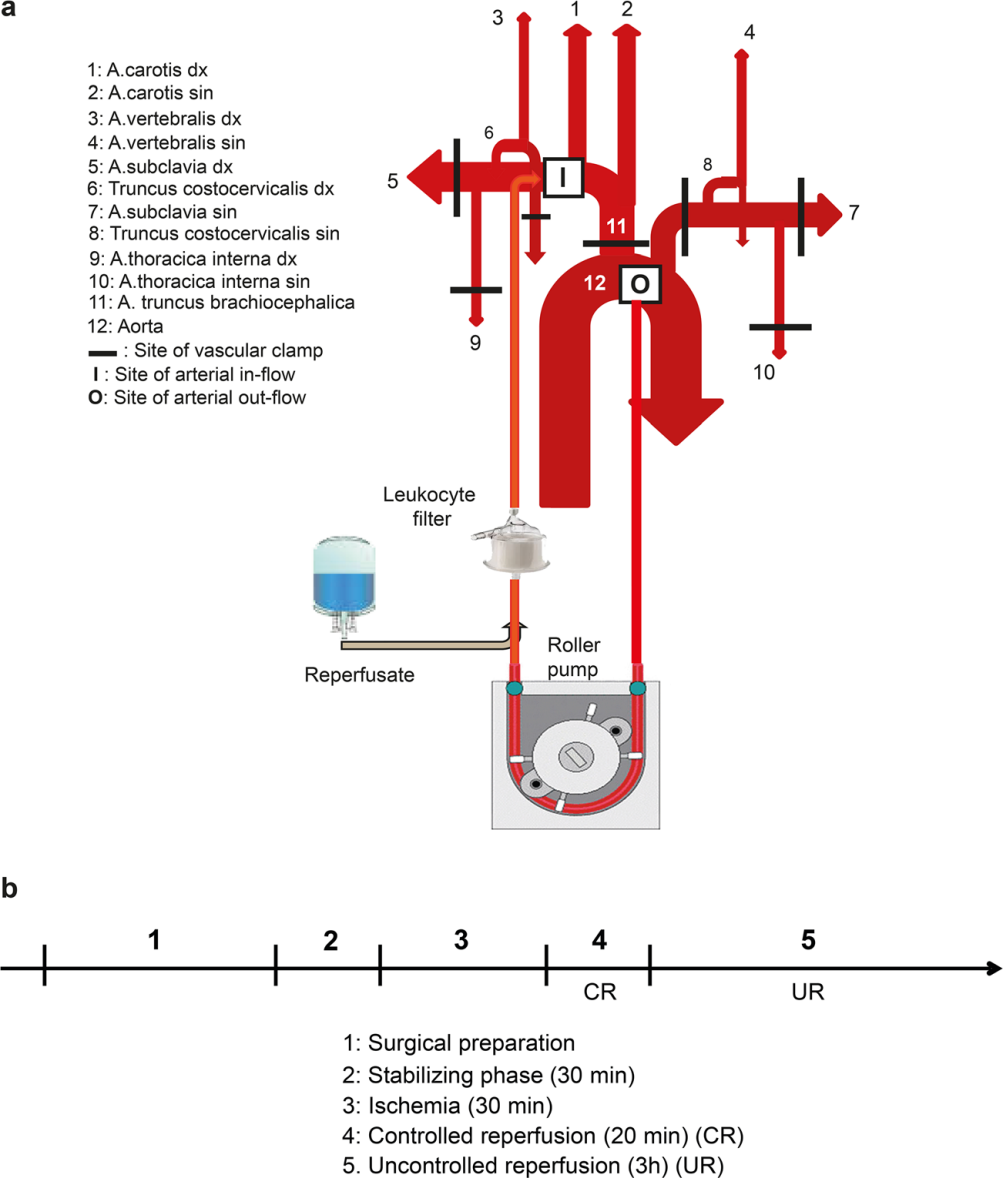
Surgical preparation, mechanical reperfusion setup, and study protocol. **a** Schematic illustration of the intrathoracic vessel anatomy, cannulation sites, and mechanical circuit setup. **b** A timeline depicting the experimental flow

The porcine anatomy varies from the human and extensive intrathoracic dissection has to be performed in order to achieve complete cerebral ischemia. After median sternotomy, all major arteries to the brain were freely dissected and prepared with vessel loops as previously described [12] and in SI. A schematic illustration is demonstrated in Fig. 1a. Global normothermic ischemia was achieved by clamping all cerebral blood supply for 30 min.

### Controlled Reperfusion

After the 30-min ischemia period, 20 min of controlled reperfusion (CR) using extracorporeal circulation was followed. All vessel clamps were left in place. Arterial drainage was from a 16-Fr cannula (Fem-Flex, Edwards Lifesciences, Irvine, CA) inserted into the distal aortic arch. A 10-Fr (Edwards Lifesciences) cannula was inserted in the right subclavian artery for arterial inflow. ECC flow was normothermic, non-pulsatile (Stöckert SIII heart-lung machine, Sorin Group Scandinavia AB, Sollentuna, Sweden) at 700–750 ml/min (15 ml/kg/min) which typically generated a mean arterial pressure (MAP) in the innominate artery of 50 mmHg. The blood was passed through a leukocyte filter (LeukoGuard LG6, Pall, Pall Norden, Lund, Sweden) placed on the arterial inflow line.

The MoRep group received the reperfusate solution coupled to the arterial inflow line via a separate pump and allowed to mix with the blood at 12–15% of the perfusion flow. This was finely tuned so that the 2 l was infused during the 20-min controlled reperfusion, averaging 100 ml/min of reperfusate to 600–650 ml/min blood, i.e., a total flow of 700–750 ml/min.

To compensate for systemic calcium losses, as a consequence of the citrate, CaCl_2_ (0.25 mmol/ml, 120 ml/h) was connected to the central venous catheter and started at the same time as the cerebral reperfusion.

Controlled cerebral perfusion lasted exactly 20 min after which the vessel clamps were removed and ordinary cerebral circulation resumed. The animals were observed for 3 h after normal circulation resumed; this phase is termed “uncontrolled reperfusion, UR” (see Fig. 1b for study protocol).

### Reperfusion Solution

The reperfusion solution was made fresh each morning, with the composition aiming to mimic the solution used in [7], as close as possible. The composition in detail: 1600 ml Ringer’s acetate (Fresenius Kabi), 300 ml ACD-A (anticoagulant citrate dextrose solution, solution A, Fresenius Kabi), 75 ml Addex-THAM (trometamol 3,3 mmol/ml, Fresenius Kabi), 20 mmol magnesium (20 ml 1 mmol/ml magnesium sulfate, Fresenius Kabi), and 500 mg thiopental (500 mg/20 ml, Pentocur, Abcur).

### Hemodynamic and Intracranial Monitoring

Central venous pressure (CVP), mean pulmonary artery pressure (pMAP), mean peripheral arterial pressure (pMAP), central pressure in the innominate artery (trMAP), intracranial pressure (ICP), and sagittal venous pressure (SVP) were monitored. Details of catheter placement can be found in SI. Heart rate and pulse oximetry (tail base of the pig) was also registered.

### Intracerebral Microdialysis

Microdialysis was performed as previously described [12] and in SI.

### Blood Gases and Oxygen Extraction Rate

Blood was drawn concomitantly from the sagittal sinus and the left superficial femoral artery. Blood gases were also drawn from the arterial inflow line in the MoRep group, distal of the buffer inflow, in order to calculate accurate oxygen extraction rates (OER), as the inflow blood was more hemodiluted than in periphery in the MoRep group given the added buffer. Cerebral OER were calculated according to [13].

### Neuropathology

The brains were harvested, fixed, and sectioned as described in SI. The brain sections were analyzed in a blinded manner. Between 2 and 7 (average 5.5), sections from each brain were examined, depending on section quality. The sections were both whole-hemispheric and smaller, on selected regions, covering significant parts of the neocortex and the hippocampus for quantitative assessment. Also, the subcortical nuclei, the white matter, and in part the cerebellum were assessed for a more qualitative evaluation of overall pathology. Pathological alterations were noted and scored according to a semiquantitative system employed in a previous study [13] and as described in SI.

### Baseline Measurements and Sham-Operated Animals

After surgical preparation, the animal was placed on their right side and allowed to recover for 30 min before obtaining baseline measurements. Heparin was given (250 IU/kg) to all three experimental groups, with the goal of maintaining activated clotting time of > 300 s. Additional doses of heparin were given as needed to maintain this.

### Statistics

For analysis of significance levels between the groups regarding the hemodynamic, blood gas and microdialysis biomarker two-way ANOVA was used, with the Bonferroni post hoc comparisons. In general, *p* < 0.05 was considered statistically significant. For the assessment of significant differences regarding neuropathological outcomes, one-way ANOVA with Bonferroni post hoc comparisons was performed. The software GraphPad Prism 7 (San Diego, CA) was used.

## Results

### Hemodynamic Analyses

**Fig. 2:**
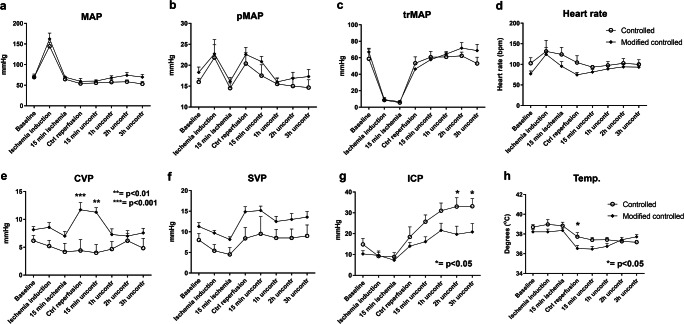
Hemodynamics. Pressure measurements in the superficial femoral artery (**a**), pulmonary artery (**b**), innominate artery (**c**), right atrium (**e**), sagittal sinus (**f**), and the lateral ventricle (**g**) all in mmHg. Pulse in beats per minute (**d**) and temperature in degrees Celsius (**h**). CVP, central venous pressure; ICP, intracranial pressure; MAP, mean arterial pressure; PA, pulmonary artery; SVP, sagittal venous pressure. *N* = 6 StRep and 7 MoRep

**Fig. 3:**
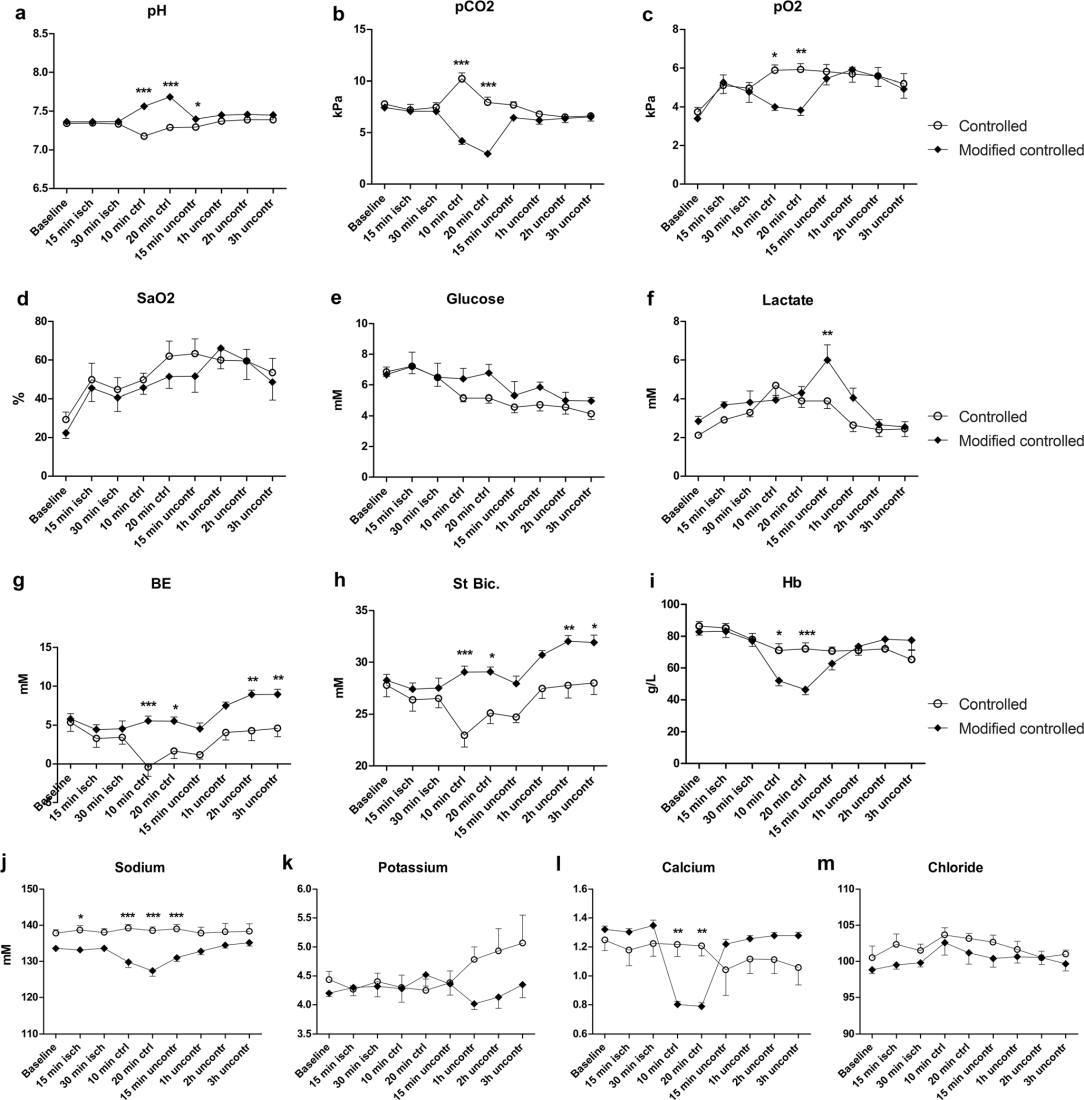
Sagittal blood gases. The pH (**a**), pCO_2_ (**b**), pO_2_ (**c**), oxygen saturation (SaO_2_) (**d**), glucose (**e**), lactate (**f**), base excess (**g**), standard bicarbonate (**h**), and hemoglobin levels (**i**), sodium (**j**), potassium (**k**), calcium (**l**), and chloride (**m**) in blood drawn from the sagittal sinus. *N* = 6 StRep and 7 MoRep

Details of hemodynamic changes are described in SI. MAP rose in both groups in conjunction with cerebral ischemia induction as a token of successful occlusion of the cerebral circulation (Fig. 2a). After the ischemic period, pressure levels were kept stable and without group differences. The pulmonary artery (pMAP) pressure was also dynamic, with a rise at ischemia induction, then a drop during ischemia (Fig. 2b). A second rise in pMAP was seen in conjunction with the start of CR, as a consequence of volume substitution, but there were no differences between the StRep and MoRep groups. The pressure in the innominate artery dropped at ischemia induction and during ischemia and then normalized, without group differences (Fig. 2c). The MAP in the innominate artery was around 50 during the CR in both groups, as was the aim. The heart rate also rose at the start of ischemia and then normalized, without group differences (not shown). The central venous pressure increased in the MoRep group during the CR and stayed elevated in the early uncontrolled phase compared with that in the StRep group, but was otherwise quite stable in all animals (Fig. 2e). The sagittal venous pressure (SVP) fell during ischemia and peaked during CR, with the same pattern and without significant differences between the groups (Fig. 2f). The ICP fell during the ischemic phase compared with baseline and then started to rise, beginning from the CR, in both groups, but rose to higher levels and continued to rise for longer in the StRep compared with that in the MoRep group (Fig. 3g). During the last 2 h of the experiment, the ICP was lower in the MoRep than in the StRep group. The temperature fell in both experimental groups at the start of CR and remained 0.5–1 °C lower than from the beginning of the experiment (not shown).

The sham animals remained largely stable in all parameters throughout the experiment, and the data is therefore not shown.

### Blood Gas and Oxygen Extraction Rate

The results from the peripheral blood gases were of less focus for the study but are for the sake of completeness described in SI and supplementary Fig. 1.

The venous gases from the sagittal sinus showed not only increased pH and decreased pCO2 but also lowered pO2, during CR in the MoRep group (Fig. 3a–c). There was no difference in saturation (SaO2) between the groups (Fig. 3d). The glucose was similar between the groups, with a gradual decrease; the lactate increased in the MoRep group after the start of uncontrolled reperfusion, but fell to similar levels as the StRep group during the latter part of the experiment (Fig. 3e and f). Both base excess (BE) and standard bicarbonate (St.bic) were higher in the MoRep group, starting from the CR, and remained so for the rest of the experiment (Fig. 3g and h). As for hemoglobin, the pattern was the same as in the periphery, with lower Hb in the MoRep group during CR, but thereafter similar between the groups (Fig. 3i). The electrolytes showed the same differences as in the periphery, except that in the sagittal sinus, there was a clear decrease in calcium, during the CR phase, as expected (Fig. 3j–m).

### Oxygen Extraction Rates

**Fig. 4:**
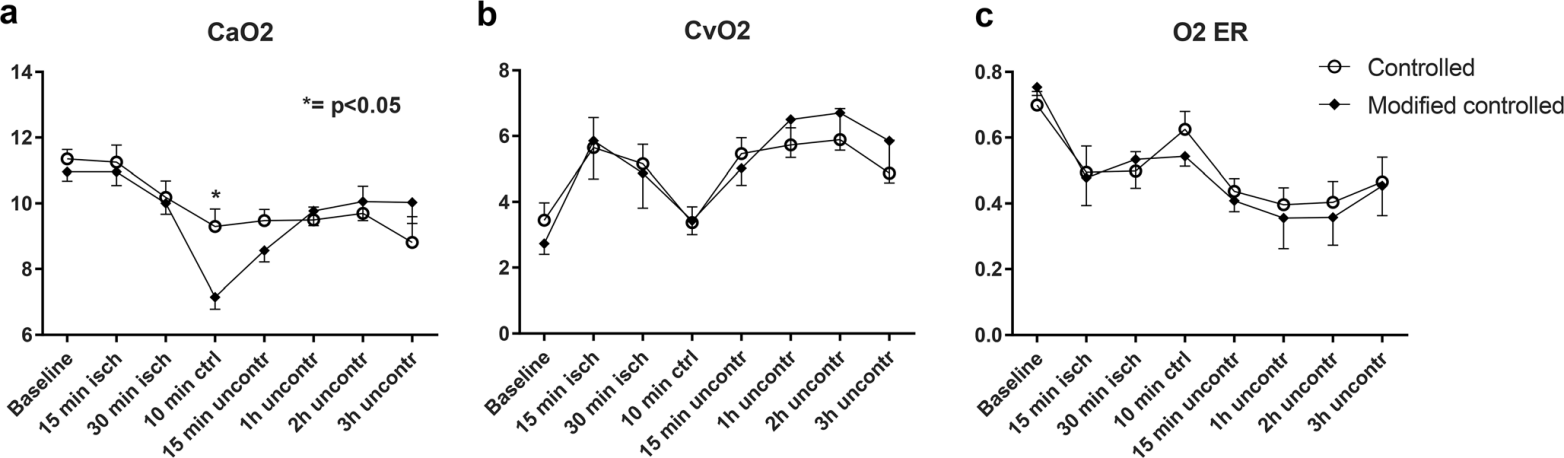
Oxygen extraction rates. No major differences in arterial (CaO_2_) (**a**) or venous (CvO_2_) (**b**) oxygen content nor oxygen extraction rates (OER) (**c**) were identified. *N* = 6 StRep and 7 MoRep

The oxygen content of the arterial blood (CaO_2_) was relatively stable during the experiment in the StRep group, but dropped in the MoRep group during the CR phase, likely as a consequence of more hemodilution (Fig. 4a). The levels of oxygen in the venous blood (CvO_2_) of the brain were more dynamic and increased during ischemia, and then dropped during the controlled reperfusion, in both groups. The CvO_2_ levels then rose again during the uncontrolled phase, but there were no differences between the groups at any phase (Fig. 4b). The oxygen extraction rates (OER) showed a general, slow decrease during the course of the experiment, with a small temporary rise during CR, but there were no significant group differences (Fig. 4c).

### Intracerebral Microdialysis

**Fig. 5:**
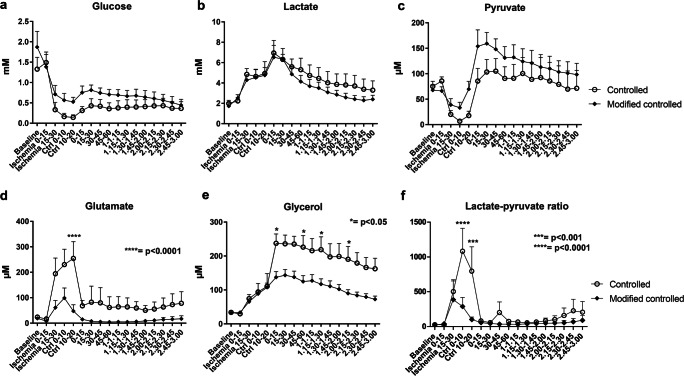
Microdialysis. Intraparenchymal measurements of glucose (**a**), lactate (**b**), pyruvate (**c**), glutamate (**d**), and glycerol (**e**). The lactate/pyruvate ratio was calculated (**f**). *N* = 6 StRep and 7 MoRep

Glucose levels showed a similar pattern in both groups, without differences, with a distinct drop during the ischemic and CR phases and then a gradual recuperation, but to lower levels than before ischemia and CR (Fig. 5a). Lactate showed an increase during ischemia and CR, with a peak during the start of return of normal circulation, but with almost identical patterns between the groups, without differences (Fig. 5b). Pyruvate also showed a dynamic, interesting pattern, again without differences between the groups, with a dip during ischemia and early CR, thereafter a steep rise during the latter part of CR and early uncontrolled reperfusion, and then values slowly declining toward the starting values (Fig. 5c). Glutamate rose in both groups during the CR, but was significantly higher in the StRep than in the MoRep group (Fig. 5d). Glycerol rose similarly in both groups during ischemia and CR. However, after CR, the curves diverged and the StRep group continued to rise in glycerol and remained elevated compared with the MoRep group during the following hours, although a slow decline in glycerol was observed in both groups during the uncontrolled phase (Fig. 5e). Urea levels were stable without group differences, indicative of reliable microdialysis function (Fig. S2).

Lactate/pyruvate (L/P) ratio showed a clear rise starting during the latter part of the ischemic period and remained elevated during the CR phase in both groups. However, the levels were greatly more elevated in the StRep compared with those in the MoRep group during the CR (Fig. 5f).

### Neuropathological Assessments

**Fig. 6:**
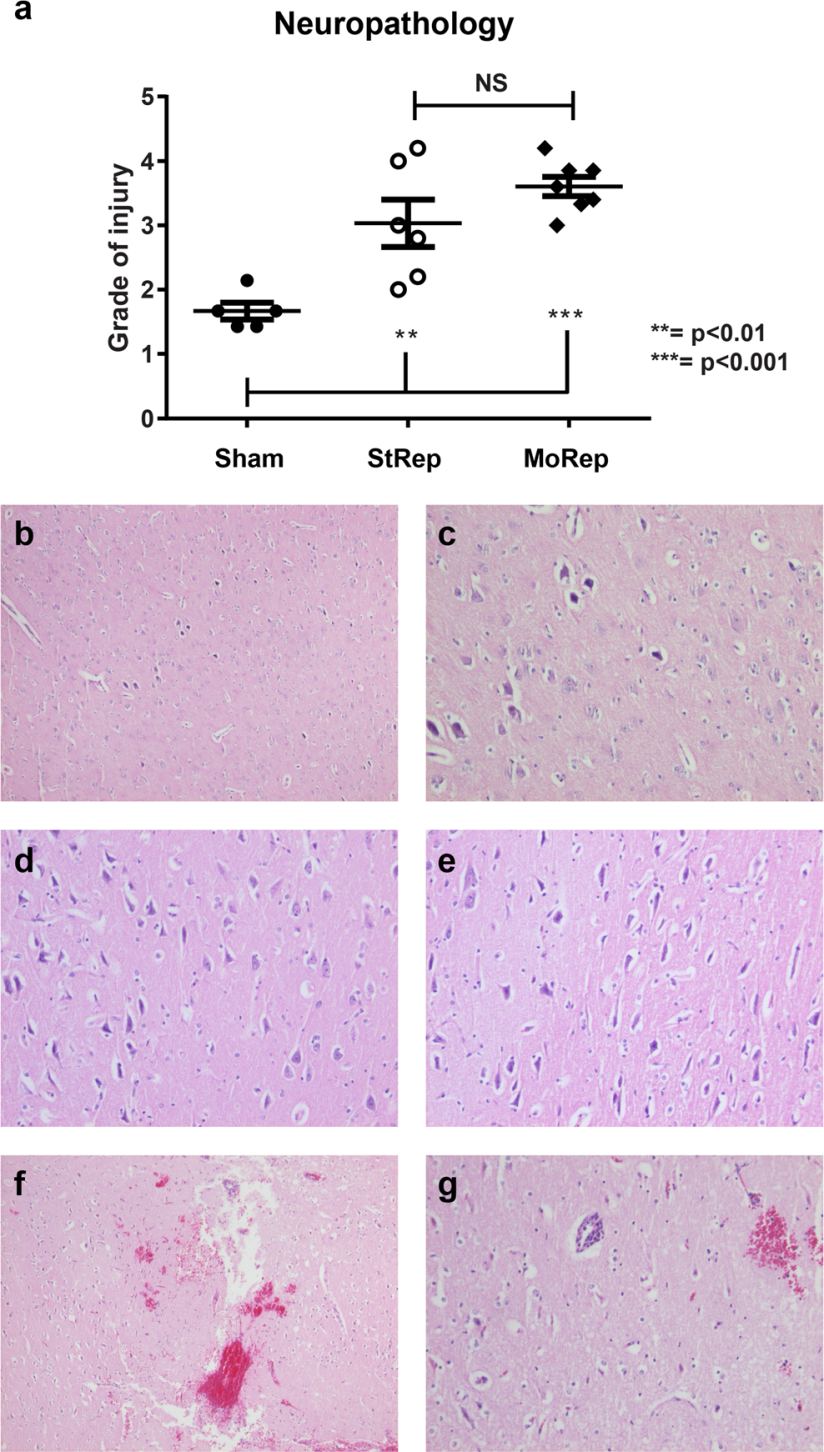
Neuropathology. The MoRep animals showed a slightly higher degree of injury than the StRep group, both differed distinctly from the sham group (**a**). Brain damage score 1: occasional shrunken and dark neurons are seen among the preserved neurons. Used objective × 4 (**b**) and × 10 (**c**). Brain damage score 2: many shrunken neurons among other preserved neurons, still the surrounding matrix is intact. Hematoxylin-eosin (HE) × 10 (**d**). Brain damage score 3: many shrunken neurons and pale vacuolated neuropil (right), delineated against preserved cortex (left). HE × 10 (**e**). Brain damage score grade 5: shrunken neurons and pale vacuolated matrix plus extravasated blood. HE × 4 (**f**) same area × 10 (**g**). *N* = 5 Sham, 6 StRep and 7 MoRep

Blinded microscopic evaluation of the sectioned pig brains demonstrated highly significantly elevated injury scores in both the ischemic groups compared with the sham-operated animals (Fig. 6a). There were no significant differences between the animals in the MoRep group and the StRep group. Illustrative sections from different degrees of injury are shown in Fig. 6b–g.

## Discussion

The present study demonstrates that a controlled, mechanical reperfusion protocol following prolonged global brain ischemia has the potential of attenuating the degree of subsequent injury. The specific aim of the current study was to further dissect and understand the innovative results achieved by [4–7], with a focus on the reperfusion solution. The addition of the buffer to the leukocyte-filtered blood proved a step forward, with improvement of multiple parameters. However, there were still clear signs of brain injury (histologically and brain swelling). Clinical short- and longer-term outcomes could not be evaluated with the current ethical permit.

One key property of the buffer was its calcium-lowering capacity, achieved by adding the ACD solution. Calcium is a major signaling molecule, not least in the CNS. An excess of calcium contributes to oxidative stress through the generation of reactive oxygen species in the mitochondria [14]. Consequently, calcium inhibition after cardiac arrest and resuscitation has proven effective to prevent brain mitochondrial injury [15]. A drawback of using ACD is that this makes the animals bleed, especially in combination with heparinization.

Another component of the reperfusion solution was thiopental. Barbiturates have been used for decades as adjunct brain protection during aortic arch surgery with circulatory arrest. Although the data is not altogether clear, a review from 2013 concludes that the use of thiopental provides additional cerebroprotection in conjunction with circulatory arrest [16].

Magnesium has been ascribed cerebroprotective effects [17] and was also supplemented to the solution. The neuroprotective mechanism is not fully known, but is likely achieved by magnesium’s role as an endogenous calcium channel antagonist at neuronal synapses, which prevents excessive activation of NMDA receptors by excitatory amino acids, such as glutamate [17]. Another protective mechanism of magnesium may be through the downregulation of inflammatory pathways [18].

The addition of THAM to the buffer avoiding a drop in pH is also important from a neuroprotective angle. The mechanism by which acidotoxicity worsens brain injury after ischemia is through acid-sensing calcium-permeable ion channels [19]. The resulting excessive calcium overload leads to calcium toxicity [20].

As calcium is such a key player in the cerebral signaling, both in normal and pathological circumstances, we removed the animal where the intended calcium-lowering was not achieved. It is unclear why the levels were not lowered in this animal. Potentially, the current study could have been even more effective to lower the calcium levels further, but this remains to be defined.

### Hemodynamics

The circulation in both experimental groups was highly dynamic, but in general without large differences between the groups. However, ICP, the most interesting measurement in the current setting, differed between the groups and was lower at the end of the experiment in the MoRep group. ICP also showed a trend of leveling out earlier than in the StRep group, where the rise continued during the course of the experiment.

### Blood Gases and OER

Most changes in the blood gases occurred during the turbulent CR phase, where especially the intended lowering of calcium in the MoRep group was evident. The addition of THAM modified pH, as planned. However, none of the observed changes translated into any differences in oxygen extraction rates, which in both groups was lower at the end than in the beginning of the experiment.

### Intraparenchymal Microdialysis

In the setting of traumatic brain injury (TBI), intraparenchymal levels of glutamate and glycerol were coupled to increased mortality [21]. Glutamate was lower in the MoRep group, especially during the CR phase, and glycerol, a possible marker of cell membrane injury [22], was also lower in the MoRep group. Also increased lactate/pyruvate ratio was coupled to increased mortality after TBI [21]. Interestingly, the MoRep group had a considerably better lactate/pyruvate ratio during the CR phase. Thus, also the microdialysis pointed to an improved milieu in the MoRep group.

### Histology

Analysis of histological sections is difficult and by necessity subject to semiquantitative scales, not always corroborated by international classification and consensus standards. The use of control and as here, lateral experimental groups, is thus essential. We could confirm that both experimental groups showed considerably more pathology than the sham animals. Interestingly, the MoRep group showed a non-significant trend of higher scores than the StRep group, which prompted a detailed analysis at the individual level. This revealed that the animal which did the worst in the StRep group (clinically brain dead with MAP equal to ICP during the last 2.5 h) had the lowest histopathological score. In line with this observation was that the second-to-worse animal in the StRep group, with ICP 40 mmHg rendering a very low cerebral prefusion pressure, also had lower histopathological scores. This could suggest that the total lack of reperfusion might have caused the pathological process to come to a halt, even though this is more speculative.

### Clinical Applications

The growing interest in E-CPR will likely render more resuscitation attempts. But as neurological outcomes still are poor after E-CPR, the methodology is in great need of improvement. The research findings herein are thus of interest, although the current model is not directly possible to translate into the setting of cardiac arrests. This, as in the situation with E-CPR, cannulation typically is performed peripherally via the femoral vessels rendering it impossible to differentiate and control the reperfusion to the brain from the rest of the body. It would therefore require perfusion of the whole body with a cerebroprotective solution during the initial phase. Even though this could be beneficial for the brain, the effect on other organs is uncertain and requires further exploration.

However, the current concept could after further evaluation and refinement be applicable during surgery of the arch in patients with aortic dissection and ongoing cerebral ischemia at the start of surgery. The direct access to the arch vessels and the use of extracorporeal circulation for selective antegrade cerebral perfusion allow the blood flow and reperfusate composition to the brain to be precisely controlled.

### Limitations

The leukocyte filters used herein were the same as in our previous study to allow for comparison. The filters should be adequate according to [23–25]; however, according to Allen/Buckberg [26], the use of a single filter may have been inadequate. It is plausible that the leukocyte filtration could become more efficient, and this should be further evaluated. One further difference in the aforementioned studies is that we have not added edarovone [7], as we could not obtain this. Edarovone has been used in experimental and clinical settings in the treatment of ischemic-injured brains [27]. Potentially, our results could have improved with the use of edarovone and several coupled leukocyte filters.

Regarding the histopathology, it is possible to argue that it may be too soon after injury to discern differences- and potential discrepancies between the experimental groups may have become more apparent after several more hours or days. Although the current studies evaluate the brain in several modalities that indeed demonstrate differences between the experimental group, none of the methods can truly evaluate neurologic function. Ideally, a neurologic status, both at wake-up and after several days, would be the ultimate outcome parameter; however, resources and ethical permits would not allow for this.

Two of the excluded animals in the MoRep group died from right heart failure due to volume overload in conjunction with the start of the CR. In later animals, this was handled by a gentler start of perfusion.

## Conclusions

There are several indicators that the current modified, controlled reperfusion protocol is beneficial compared with reperfusion with only leukocyte-filtered blood, as well as compared with sheer uncontrolled reperfusion, following global brain ischemia. Even though the protocol is insufficient in fully preventing brain injury from developing, the results constitute a real progress in the establishment of a cerebral reperfusion strategy following global brain ischemia.

## Electronic Supplementary Material

ESM 1 (DOCX 30 kb).

ESM 2 (PNG 1074 kb).

High Resolution Image (TIF 1007 kb).

ESM 3 (PNG 146 kb).

High Resolution Image (TIF 201 kb).

## Abbreviations

CR: Controlled reperfusion
MoRep: Modified controlled reperfusion group
StRep: Standardized controlled reperfusion group
UR: Uncontrolled reperfusion
