# Supplementary material for: Mechanical Reperfusion Following Prolonged Global Cerebral Ischemia Attenuates Brain Injury
Source: J Cardiovasc Transl Res. 2020 Jul 17;14(2):338–47. doi: 10.1007/s12265-020-10058-9 (PMC8043930; doi:10.1007/s12265-020-10058-9)
Supplement: Supplementary file 1 — (DOCX 30 kb). [file 12265_2020_10058_MOESM1_ESM.docx]

# Supplemental Information

**Title: Mechanical reperfusion following prolonged global cerebral ischemia attenuates brain injury**

**Short title: Mechanical reperfusion after global brain ischemia**

Rickard P.F. Lindblom MD, PhD^1,2^, Thomas Tovedal PhD^1,3^, Bo Norlin M.Sc ^1^, Lars Hillered MD, PhD^4^, Elisabet Englund MD, PhD ^5^, Stefan Thelin MD, PhD ^1,2^

1) Department of Cardiothoracic Surgery and Anesthesia, Uppsala University Hospital, Sweden

2) Department of Surgical Sciences, Section of Thoracic Surgery, Uppsala University

3) Department of Surgical Sciences, Section of Anesthesiology and Intensive Care, Uppsala University.

4) Department of Neuroscience, Neurosurgery, Uppsala University

5) Department of Clinical Sciences, Lund University, Lund

**Correspondence:**

Rickard P.F. Lindblom

Cardiothoracic Surgery and Anesthesia

Uppsala University Hospital

SE-751 85 UPPSALA

Sweden

[Rickard.lindblom@surgsci.uu.se](mailto:Rickard.lindblom@surgsci.uu.se)

## Results

Clinical management, hemodynamic and perfusion measurements

The circulation was during the course of the experiments highly dynamic, with an massive rise in blood-pressure and heart rate, as a consequence of the endogenous amine surge in conjunction with closing the cerebral circulation. This was handled with infusions of Esmolol 10mg/ml (7-8ml/h) or Nitroprusside 1mg/ml (7-8ml/h). During other phases of the experiment, for instance at the start of the controlled reperfusion, where a substantial blood volume was drained into the extracorporeal circuit, the animals apart from volume substitution (Ringer’s Acetate) often needed intermittent doses of amines like Ephedrine (5mg/ml) or Phenylephrine (0.1mg/ml) to maintain mean arterial blood pressure (MAP) above 40-50mmHg. A blood pressure drop was also often seen after start of uncontrolled reperfusion when the blood circulation again quickly was redistributed to the brain, now without mechanical support.

*Peripheral blood gases*

Blood gases from the superficial femoral artery showed increased pH and lower pCO2 during the controlled reperfusion (CR) phase in the MoRep group, as a consequence of the THAM-buffert (**Fig. S1a+b**). There were no differences in pO2 during the initial or controlled phase of the reperfusion, however, during the uncontrolled reperfusion phase, pO2 was higher in the StRep group, suggesting lower oxygen consumption in the StRep than in the MoRep group (**Fig. S1c**). There were no differences in glucose levels, with a similar decline in both groups during the course of the experiments, however, there was a significant lactate rise in the MoRep group during the late phase of the CR and early in the uncontrolled reperfusion phase (**Fig. S1e+f**). As expected, there was a drop in hemoglobin in the MoRep group during the controlled phase, not surprising as 2 liters of fluid were infused during the 20 minute CR phase (**Fig. S1i**). However, also the StRep group required volume transfusion during the CR to substitute the volume circulating extra-corporeally, but were given less than the MoRep, which explains the Hb drop also in the StRep group. The sodium levels were for some reason higher in the StRep group, already from the start of the experiments, but were for both groups within normal references except during the latter part of the CR in the MoRep group (**Fig. S1j**). The potassium levels were quite dynamic, but only significantly different between the groups during the last hours, when they rose in the StRep group (**Fig. S1k**). The systemic calcium levels showed a tendency to drop during the CR, in spite of calcium substitution, but the differences between the groups was not significant (**Fig. S1l**). Chloride levels remained stable and similar (**Fig. S1m**).

## Materials and Methods

Anesthesia, general preparations and euthanasia

To induce anesthesia a subcutaneous injection of a mixture of xylazine (Rompun® 2.2 mg/kg, Bayer A/S, Lyngby Denmark) and tiletamine/zolazepam (Zoletil 100® 6.0 mg/kg, Virbac S.A., Carros, France) was used. A buffered glucose solution carrier (Rehydrex® with glucose 25 mg/ml, Fresenius Kabi AB, Uppsala, Sweden) with ketamine 30 mg/kg/h (Ketaminol Vet® 100 mg/ml, Intervet AB, Stockholm, Sweden), fentanyl 0.04 mg/kg/h (Fentanyl 50 μg/ml, B BraunMedical AB, Danderyd, Sweden), midazolam 0.1 mg/kg/h (Midazolam Hameln 1 mg/ml, AlgolPharma AB, Kista, Sweden) and rocuronium bromide 0.3 mg/kg/h (Esmeron ® 10 mg/ml, Merck Sharp & Dohme AB, Sollentuna, Sweden) was used to maintain anesthesia. The pigs were intubated and normoventilated using volume control with 40% O_2_ in a Siemens Servo-I ventilator (Maquet Co. KG, Rastatt, Germany), with end-tidal CO_2_ kept between 4.6 and 5.1 kPa.

Cefuroxime 750mg was given intravenously before the first skin incision, and a second dose was given after three hours. The urinary bladder was identified intraabdominally and a urinary catheter secured into the bladder with a purse string suture. During the whole experiment the animals were kept on a heating mattress with the temperature of 39^○^C.

In general, minimal interference was made to adjust blood glucose, which in all pigs dropped during the experiment, unless below 3 mmol/L in which case a bolus injection of glucose 300mg/ml was given.

At the end of the experiment a lethal dose of potassium was injected intracardially and 1000ml of 4^○^C NaCl was infused in the in-flow cannula in the subclavian (see manuscript) at 300ml/min to cool the brain and wash out blood for optimal storage and sectioning. A vascular clamp was placed proximally of this cannula to avoid more blood reaching the brain. A second 16Fr cannula (Fem-Flex, Edwards Lifesciences, Irvine, CA) was inserted into superior vena cava and connected to a suction device for drainage of blood and the infused NaCl to avoid cerebral congestion during the NaCl perfusion.

Surgery and isolation of cerebral blood-flow

After median sternotomy all major arteries to the brain were freely dissected and prepared with rubber vessel loops to enable easy clamping when ischemia was induced. In summary: vessel clamps were placed on the right subclavian artery distal of the right internal thoracic artery (RITA) and proximally on the innominate to occlude arterial flow to the brain from both the carotid arteries, which both derive from the innominate artery, and the right vertebral artery. The RITA was ligated and divided. To isolate the right vertebral artery and prevent collateral flow to the brain via this route, vessel clamps had to be placed on the right costocervical trunk, which originates posteriorly from the subclavian artery, distal of the common carotid trunk and proximally of the RITA. On the left side, clamps were placed proximally on the left subclavian artery, close to the aorta to exclude collateral flow via the left costocervical trunk (from which the left vertebral artery originates) which arises dorsally from the subclavian artery. A clamp was also placed distally of the left internal thoracic artery (LITA) to occlude retrograde cerebral inflow to the costocervical trunc/vertebral. The LITA was ligated and divided. A summary is shown in figure 1.

The sham-operated animals had sternotomy and full surgical preparation intrathoracically, but no ischemia was induced. In a previous study also sham operated animals, that had intracranial catheters placed and were fully heparinized often developed some intracranial bleeding and ICP rises [1]. For that reason, no intracranial catheters were placed the sham animals in the current study, and these animals mainly served as a source of brain tissue. They were observed for an equal period of time as the ischemic groups and received the same anesthesia, euthanasia and brain harvesting protocol.

Hemodynamic and intracranial monitoring

Central and peripheral hemodynamic parameters were continually measured. In brief a three-lumen central venous catheter was placed in the right external jugular vein, into which also a Swan-Ganz catheter was placed and introduced into the pulmonary artery. A pressure catheter also used for obtaining blood gases was placed into the left superficial femoral artery. A second arterial line was placed in the innominate artery.

With the animals in a prone position a midline incision on the head was performed and the intersection between the sagittal and the fronto-parietal suture was identified. A drill hole (1.5 cm in diameter) was made here and the sagittal sinus identified, into which a pressure catheter was inserted and connected to a 3-way stop-cock for blood sampling and sagittal venous pressure (SVP) measurements.

1.5 cm laterally to the right along the frontal-parietal suture, 2.5 cm anteriorly of the suture another hole was drilled. The dura was opened and a ventricular drain was placed into the lateral ventricle, with clear cerebrospinal fluid in exchange confirming the position. The drain was connected to a pressure set for continuous intra-cranial pressure (ICP) measurements.

Intracerebral microdialysis

The analytes were used as biomarkers of energy crisis (glucose, lactate, pyruvate, lactate/pyruvate ratio), excitotoxicity (glutamate) and cell membrane degradation/oxidative stress (glycerol) [2-4]. Urea was analysed as an internal control.

With the animal still in prone position, a small hole was drilled 1.5 cm laterally to the left and 1.5 cm frontal of the intersection between the sagittal and the fronto-parietal suture. A hollow bolt (GSM Licox, Germany) was placed in the hole into which a microdialysis catheter (70 Microdialysis Bolt Catheter; M Dialysis AB, Stockholm, Sweden) with 10 mm polyamide membrane and a 20 kDa cut off (M Dialysis AB, Stockholm, Sweden) was inserted into the cerebral parenchyma. The catheter was perfused with microdialysis fluid (Perfusion Fluid T1, containing NaCl 147mM, KCl 4mM, CaCl_2_ 2.3mM, M Dialysis AB) at a rate flow rate of 1µL/min using a 106 MD pump (M Dialysis AB). The dialysate was continuously collected in vials, harvested one-by-one at 15minute intervals. The samples were analyzed in ISCUSflex analyzer (M Dialysis AB) for glucose, lactate, pyruvate, glutamate, glycerol and urea. Lactate/pyruvate ratio was calculated. Quality controls at two different concentrations for each analyte were performed every weekday. Imprecision values for between assay co-efficient of variation was < 10% for all analytes. Urea was used as an endogenous control for microdialysis catheter performance [5].

Neuropathological assessment of the brain

Immediately after euthanasia the skull was opened, the brain separated from the spinal cord and carefully removed. The left hemisphere was taken *en bloc* and placed into 4% paraformaldehyde for one month of fixation. Subsequently the fixed hemisphere was cut into 8 – 10 coronal slices (approximately 1 cm thick) and embedded in paraffin following standard procedure applied for human brain. Seven micrometer sections were cut and stained with hematoxylin and eosin (HE) stain and assessed under light microscopy.

Pathological alterations were noted and scored according to a semiquantitative system employed in a previous study (13) for several different types of alterations and also for sum of alterations and total degree of severity. These alterations were pycnotic/shrunken neurons, sometimes with a perineuronal halo, areas of pale and vacuolated neuropil, and regions of extravasated blood. These alterations were seen to concur progressively, i.e. the latter lesions were in general accompanied by the former, while pycnotic neurons were at times seen to occur as the sole change. The named lesions were scored 1 to 5 in each section: 1 for occasional shrunken neurons, 2 for numerous shrunken neurons, 3 for small and occasional areas of pale vacuolated neuropil, 4 for large areas of vacuolated neuropil and 5 for extravasated blood. The sum of section scores was divided by the number of assessed sections and hence each brain was assigned a mean sum score between 0 and 5, indicating all from no damage to severe hypoxic-ischemic brain damage.

## Figure legends

**Supplementary figure 1. Femoral blood gases.**

The pH (a), pCO_2_ (b), pO_2_ (c), oxygen saturation (SaO_2_) (d), glucose (e), lactate (f), base excess (g), standard bicarbonate (h), hemoglobin levels (i) sodium (j), potassium (k), calcium (l) and chloride (m) in blood drawn from the superficial femoral artery. N=6 StRep and 7 MoRep.

**Supplementary figure 2. Microdialysis- urea.**

Urea levels were stable throughout the experiment without group differences. N=6 StRep and 7 MoRep.

## References

[1] Lindblom RP, Tovedal T, Norlin B, Hillered L, Popova SN, Alafuzoff I *et al.* *Mechanical reperfusion with leucocyte-filtered blood does not prevent injury following global cerebral ischaemia*. Eur J Cardiothorac Surg 2016.

[2] Marklund N, Salci K, Lewen A, Hillered L. *Glycerol as a marker for post-traumatic membrane phospholipid degradation in rat brain*. Neuroreport 1997;**8**:1457-61.

[3] Lewen A, Hillered L. *Involvement of reactive oxygen species in membrane phospholipid breakdown and energy perturbation after traumatic brain injury in the rat*. J Neurotrauma 1998;**15**:521-30.

[4] Hutchinson PJ, Jalloh I, Helmy A, Carpenter KL, Rostami E, Bellander BM *et al.* *Consensus statement from the 2014 International Microdialysis Forum*. Intensive Care Med 2015.

[5] Ronne-Engstrom E, Cesarini KG, Enblad P, Hesselager G, Marklund N, Nilsson P *et al.* *Intracerebral microdialysis in neurointensive care: the use of urea as an endogenous reference compound*. J Neurosurg 2001;**94**:397-402.
