# Supplementary figures and images for: Mechanical Reperfusion Following Prolonged Global Cerebral Ischemia Attenuates Brain Injury
Source: J Cardiovasc Transl Res. 2020 Jul 17;14(2):338–47. doi: 10.1007/s12265-020-10058-9 (PMC8043930; doi:10.1007/s12265-020-10058-9)

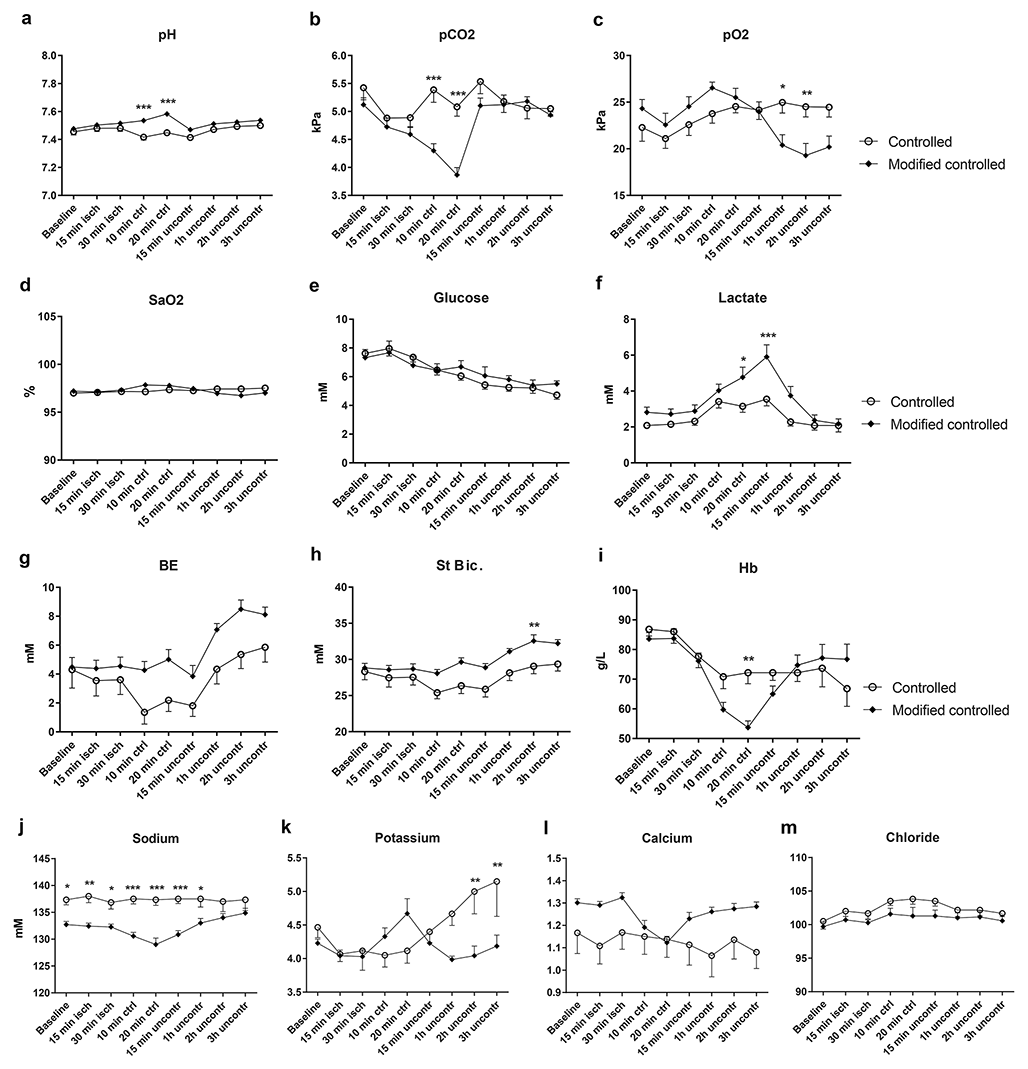

Supplement: Supplementary file 2 — (PNG 1074 kb). [file 12265_2020_10058_Fig7_ESM.png]

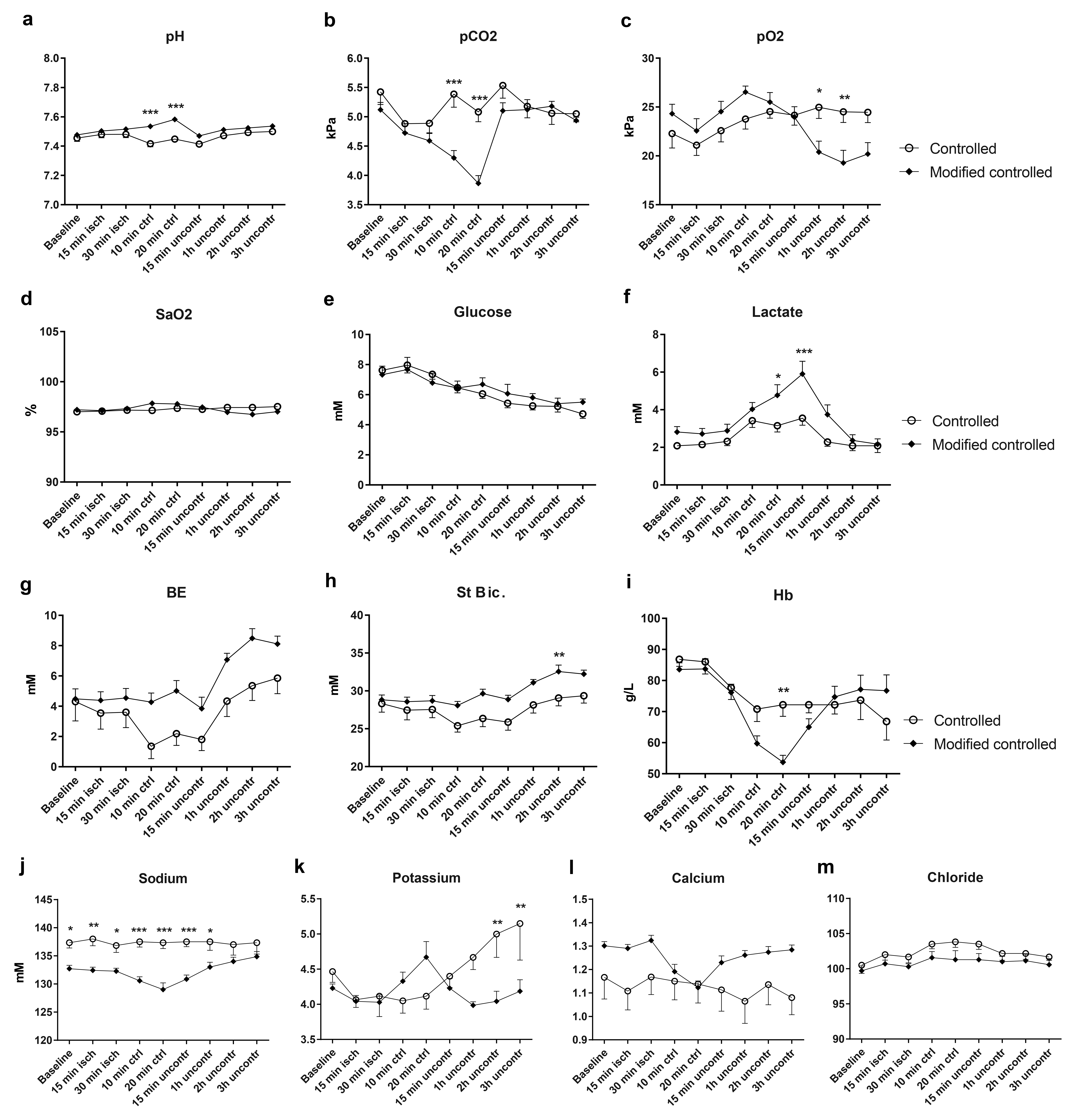

Supplement: Supplementary file 3 — High Resolution Image (TIF 1007 kb). [file 12265_2020_10058_MOESM2_ESM.tif]

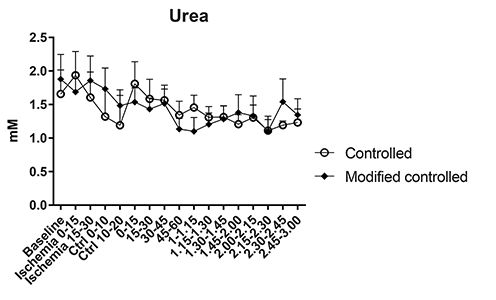

Supplement: Supplementary file 4 — (PNG 146 kb). [file 12265_2020_10058_Fig8_ESM.png]

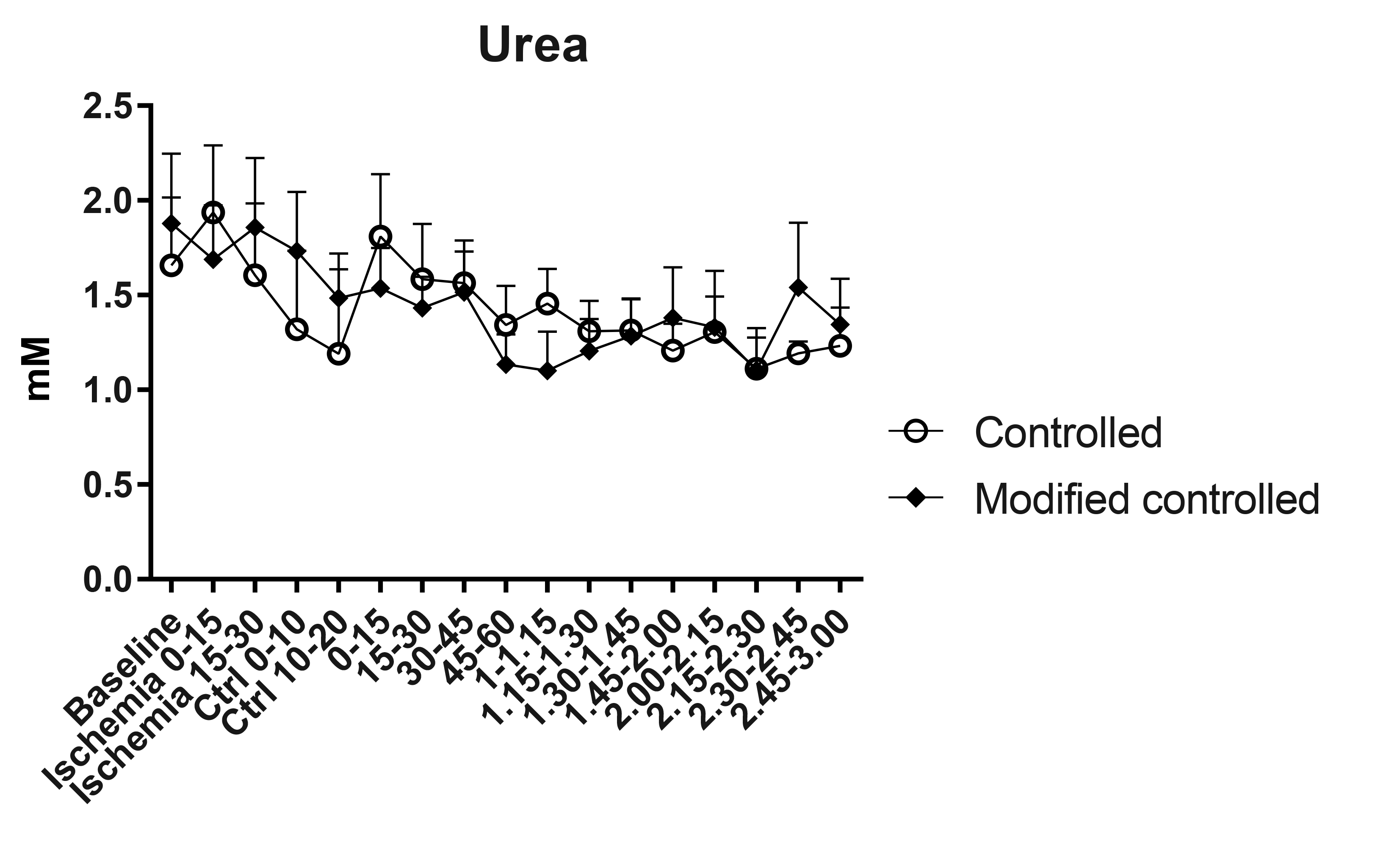

Supplement: Supplementary file 5 — High Resolution Image (TIF 201 kb). [file 12265_2020_10058_MOESM3_ESM.tif]
